# Diet Quality for Sodium and Vegetables Mediate Effects of Whole Food Diets on 8-Week Changes in Stress Load

**DOI:** 10.3390/nu10111606

**Published:** 2018-11-01

**Authors:** Hoda Soltani, Nancy L. Keim, Kevin D. Laugero

**Affiliations:** 1 Department of Nutrition, University of California, Davis, CA 95616, USA; hsoltani@ucdavis.edu (H.S.); nancy.keim@ars.usda.gov (N.L.K.); 2 Obesity and Metabolism Research Unit, USDA/ARS/Western Human Nutrition Research Center, Davis, CA 95616, USA

**Keywords:** diet, diet quality, allostatic load, perceived stress, cortisol, dietary guidelines

## Abstract

Very little is known about how whole food diets, such as those based on the Dietary Guidelines for Americans (DGA), influence psychological stress and physiological stress load. To better understand the effects of whole food diets on stress, we examined in a randomized control trial the effects of a DGA-based diet on markers of psychological and physiological stress. A randomized, double-blind, controlled 8-week intervention was conducted in overweight and obese women randomly assigned to one of two diet groups: a diet based on the 2010 DGA or a diet based on a Typical American Diet (TAD). The Perceived Stress Scale and allostatic load were used to assess stress load. Eight-week change in perceived stress did not significantly (*p* = 0.45) differ between the DGA (+0.53 ± 0.99) and TAD (−0.57 ± 0.99) groups. Likewise, 8-week change in allostatic load did not significantly (*p* = 0.79) differ between the two diet intervention groups (DGA: +0.001 ± 0.26 vs. TAD: +0.105 ± 0.28). However, we did find strong inverse associations between 8-week change in stress and intervention-based improvements in diet quality (lower sodium and higher vegetable consumption). When statistically accounting for these inverse associations, we found that perceived stress and allostatic load were higher (*p* < 0.04) in the DGA group. These findings suggest that improvements in dietary vegetable and sodium intake mediated effects of the diet intervention on psychological and physiological stress load. That is, adopting and adhering to a diet of higher quality (DGA) for 8 weeks may have been generally more stressful in the absence of improvements in vegetable or sodium consumption. This study provides further evidence for the mental health benefits of maximizing vegetable and minimizing sodium consumption.

## 1. Introduction

It is generally accepted that a healthy diet can help manage stress and prevent stress-related diseases such as depression. However, evidence-based, specific dietary recommendations that address this issue do not exist. Nutritional medicine in psychiatry is an emerging field [1], and research-driven information on stress, mood, and nutrition interrelationships is increasing. There are studies showing that nutrients and specific foods can influence an individual’s physiological, neural, and psychological response to stress [2,3,4,5,6,7,8,9,10,11,12,13,14,15,16]. For example, high-fat meals were shown to exacerbate detrimental autonomic nervous system and cardiovascular responses to stress [8], while increases in polyunsaturated fats reduced these stress-induced cardiovascular responses [3]; omega-3 fatty acid consumption normalized abnormally low cortisol responses to an acute stress test [6]; consuming milk-based phospholipids were also shown to improve memory in men reporting high levels of chronic stress [14]; and consuming egg powder was demonstrated to normalize the neuroendocrine response to stress and reduce adaptation to acute stress by normalizing the endocrine and the negative emotional response to stress [15]. Finally, it was shown that consumption of fermented foods is associated with reduced social anxiety [17]. However, very little is known about how whole food diets, such as those based on the Dietary Guidelines for Americans (DGA), influence perceived stress and overall chronic stress load.

While there is potential for whole food diets, such as those recommended by the DGA, to mitigate unhealthy stress reactions and prevent detrimental effects of stress, DGA adherence is generally low [18,19]. The cardiometabolic benefits of shifting to a healthy diet are often fleeting due to dietary and weight relapse [20]. Those who can durably adopt DGA recommended diet patterns, however, have reduced risk for developing obesity-related diseases. Studies in type 2 diabetics have shown the effects of diet and weight loss programs to be limited by dietary relapse and weight regain over the long term [21,22,23]. Increased risk for dietary and weight relapse is thought to be, in part, mediated by neurological factors linked to psychological, emotional, and cognitive function [24,25,26,27]. For example, experiencing stress during or in response to dieting or adopting new dietary patterns may significantly impede substantial progress in making these dietary changes durable [28,29], consequently limiting the overall utility of dietary recommendations. 

Psychological or mental stress can increase unhealthy food choices, impede weight loss, and promote metabolic dysfunction [30]. Stress can increase motivation to consume highly palatable, calorically dense food and, at the same time, disrupt cognitive functions associated with self-regulation and goal-directed decision-making [31]. Reward-based eating and eating as a reaction to external cues are enhanced by stress and can override homeostatic regulation of food intake [32,33]. Self-regulation and the ability to mindfully weigh short-term reward versus long-term consequences are cognitive processes thought to be important to sustaining healthy changes in eating habits, particularly in the face of stress [34]. Given the present day preponderance of societal stress [35,36] and overindulgence of highly accessible palatable, low-quality diets [37,38], a better understanding of stress networks controlling motivation, eating behavior, decision making, self-regulation, and psychological wellbeing will help to inform on the likelihood of adopting meaningful and lasting changes in diet, and highlight new and key target areas for improving nutrition and disease prevention messaging.

To better understand the effects of whole food diets on chronic stress load, we examined in a randomized control trial the effects of a DGA-based diet on chronic stress load, measured as self-reported perceived stress and allostatic load. Exploratory analyses were also performed to assess if the magnitude of departure from the participants’ usual pre-intervention diet quality mediated the effects of the intervention diet on perceived stress and physiological stress load. A primary report on the effect of the intervention on clinical risk factors for type 2 diabetes and cardiovascular disease was recently published [39].

## 2. Materials and Methods

### 2.1. Study Design

A detailed description of the study design was reported by Krishnan et al. [39]. This report is a secondary analysis based on the women who participated in the primary study examining the effects of whole food diets on cardiometabolic risk factors. In the primary study, and hence in this ancillary study, a randomized, double-blind, controlled 8-week intervention was conducted in overweight and obese women randomly assigned to one of two diet groups: a diet based on the 2010 Dietary Guidelines for Americans (DGA) [40] or a diet based on a Typical American Diet (TAD). Both diets were designed to maintain body weight over the 8-week trial and provide foods and beverages commonly available to consumers. The DGA diet contained more whole grains, low-fat dairy, polyunsaturated fat, vegetables, and fruit, while the TAD included more added sugars, refined grains, and solid fats. A specific nutrient breakdown, sample menus, and key products used to prepare meals were previously published [39]. The trial was conducted at the Western Human Nutrition Research Center in Davis, CA. The study was approved by the University of California, Davis Institutional Review Board and informed consent was given by subjects prior to participating in the study. Study participants were informed that the research was investigating a broad set of factors that may contribute to understanding dietary effects on the body. Following orientation to the study, subjects were examined over a 1-week baseline (pre-intervention) period during which they continued to consume their usual diets. This pre-intervention period was followed by the 8-week, meal-controlled diet intervention. During the pre-intervention period, physical activity, usual diet, energy requirements, body composition, cardiometabolic risk markers, and markers of psychological and physiological stress were estimated. These measurements were repeated at the end of the 8-week intervention. A detailed description of the study timeline and measurements performed in this study can be found in the publication by Krishnan et al. [39]. This trial was approved by the University of California, Davis Institutional Review Board and is registered (NCT02298725) at clinicaltrials.gov. All participants provided written informed consent for participating in the study.

### 2.2. Subjects

Overweight or obese women, ages 20–64, with a BMI of 25–39.9 kg/m^2^, and who displayed either insulin resistance and/or dyslipidemia and did not meet the minimal physical activity guidelines of 150 min/week were recruited for the study. Eligibility for the screening process was determined by obtaining information provided in a telephone screening questionnaire. Exclusion criteria included the following: presence of any metabolic diseases, gastrointestinal disorders, cancer, or other serious chronic disease; pregnancy or lactating; current use of tobacco; prescribed or over-the-counter weight-loss medications in the 6 months before enrollment into the study; moderate or strenuous physical activity >30 min/day on ≥5 days/week; weight change of >5% of body weight within 6 months of entry into the study; resting blood pressure >140/90 mmHg, hemoglobin <11.5 g/dL, total cholesterol >300 mg/dL, LDL cholesterol >189 mg/dL, triglycerides >400 mg/dL, clinically abnormal thyroid or liver function; “graveyard” work shifts or forced to stay awake all night; dietary restrictions that would interfere with consuming the intervention foods; or use of corticosteroids and medications for elevated lipids or glucose. Vulnerable populations, including adults unable to consent, infants, children, and prisoners were considered ineligible for the study.

### 2.3. Measurements

#### 2.3.1. Dietary Measurements

To estimate pre-intervention dietary habits, 24-h dietary recalls were conducted unannounced and recorded three times during the pre-intervention period. On each of the 3 days, an automated and self-administered system [41] was used by participants to input their intake for the previous 24 h and then used to assess their typical intake. Once entered, dietary data were quantified into specific nutrient and food categories and used to estimate participants’ Healthy Eating Index (HEI) scores [42,43]. The HEI is an estimate of diet quality based on the Dietary Guidelines for Americans. HEI scores for 12 different dietary components, including empty calories, fats, total protein, total vegetables, total fruits, greens and beans, whole fruits, refined grain, whole grain, seafood and plant protein, and sodium, were derived and also summed to yield a total HEI score, which has a maximum value of 100. A higher score indicates higher dietary quality and greater adherence to the Dietary Guidelines for Americans.

#### 2.3.2. Stress Measurements 

Allostatic load (AL) is an objective index of chronic stress that incorporates subclinical measures (e.g., hsCRP) across a range of multiple biomarkers that interact with activity in stress pathways including the sympathetic nervous system (catecholamines, epinephrine, and norepinephrine) and the hypothalamic–pituitary–adrenal axis (cortisol) [44,45]. AL predicts a broad range of stress-related diseases or conditions and may be better than traditional methods [46]. Allostatic load is a construct used to describe the aggregate burden that results from the body’s adaptation to the physiological dysregulations caused by life stress [47,48,49,50]. Specifically, the activation of hormonal mediators of the stress response, glucocorticoids and catecholamines, results in residual damage of the body [51,52]. This study used the following measures to determine participants’ allostatic load: 12-h urinary cortisol (corrected for urinary creatinine levels); 12-h urinary epinephrine (corrected for urinary creatinine levels); 12-h urinary norepinephrine (corrected for urinary creatinine levels); systolic and diastolic blood pressure; waist:hip ratio; HDL cholesterol; total cholesterol; dehydroepiandrosterone sulfate; and HbA1c. AL was calculated using the method reported by Gallo et al. [53], which also takes into account hyper- and hypocortisolemia, as estimated by cortisol concentrations falling into the highest or lowest octiles (12.5%) of the study sample. Subjects having cortisol concentrations in the upper or lower octiles were taken to indicate a higher allostatic load contribution by this neuroendocrine parameter. Cutoffs for all other allostatic load markers were determined by using the sample population quartiles for each marker.

To examine perceptions of stress, subjects filled out the 10 item Perceived Stress Scale. The Perceived Stress Scale-10 (PSS-10) is a validated 10-item questionnaire-based measure of perceived stress that has been well documented in the literature [54,55,56]. The PSS is intended to assess how unpredictable, uncontrollable, and overloading participants perceive their lives over the past month [57]. Subjects responded on a 5-point scale ranging from 0 (never) to 4 (very often), with higher total scores indicating higher levels of perceived stress. The questionnaire was designed for use with community samples with at least a junior high school education or equivalent [54].

#### 2.3.3. Physical Measurements

Anthropometric measurements consisted of height, weight, and waist-to-hip ratio measurements. Body weight was measured to the nearest 0.1 kg using a calibrated electronic scale (Tanita BWB-627A Class III electronic scale; Toledo Scale, Mettler-Toledo, LLC, Columbus, OH, USA) with subjects wearing lightweight surgical scrubs. Height was measured to the nearest 0.1 cm using a wall-mounted stadiometer (Model S100; Ayrton Corporation, Prior Lake, MN, USA). Body Mass Index (BMI) was calculated as kg/m^2^. Waist and hip circumferences were measured in duplicate with an anthropometric tape. Waist circumference was measured as the minimum circumference between the iliac crest and the rib cage, and hip circumference was measured at the maximum protuberance of the buttocks. Blood pressure was measured using a standard blood pressure cuff (GE DINAMAP vitals monitor; GE Healthcare, Chicago, IL, USA) placed on one arm.

### 2.4. Bio-Specimen Collection and Biochemical Assays

Fasting blood samples were collected in the morning in Vacutainers (Becton Dickinson VACUTAINER Systems, Rutherford, NJ, USA) prepared for serum and plasma (EDTA). Serum vacutainers were allowed to clot at room temperature for 30 min prior to centrifugation at 1300× *g* at 4 °C for 10 min; plasma vacutainers were kept on ice directly after collection and centrifuged at 1300× *g* at 4 °C for 10 min. Whole blood was aliquoted from the EDTA tube prior to centrifugation and stored at −80 °C for analysis. Serum and plasma aliquots were stored at −80 °C for analysis. Urine was collected for a period of 12 h, starting the evening prior to the subject’s lab visit and after consuming a controlled dinner meal, and ending with the first urinary void in the morning. Urine aliquots and acidified urine aliquots (30 mM HCl) were stored at −80 °C for analysis.

Serum HDL, LDL, and total cholesterol analyses were conducted using Beckman Coulter DxC 600/800 platform using Beckman Coulter reagent kits. Serum DHEA-S was analyzed on a Cobas e411 immunoassay analyzer (Roche Diagnostics, Indianapolis, IN, USA). Plasma CRP was analyzed on the MSD Sector Imager 2400 using the Vascular Injury Panel 2 reagent kit (Meso Scale Discovery, Rockville, MD, USA). Whole blood hemoglobin A1c (HbA1c) and urine creatinine were analyzed on the Cobas Integra 400 Plus Analyzer (Roche Diagnostics, Indianapolis, IN, USA). Urine cortisol was analyzed by ELISA (Alpco, Salem, NH, USA), and urine catecholamines were analyzed by ELISA (Bi-Cat ELISA, DLD, Hamburg, Germany).

### 2.5. Statistical Analysis 

Statistical analyses were performed using SAS for Windows, release 9.4 (Cary, NC, USA). Tests for main effects of diet intervention group (DGA vs. TAD) on 8-week change in perceived stress scale score (*N* = 22 for DGA and 22 for TAD) and allostatic load (*N* = 22 for DGA and 19 for TAD) were performed using the general linear models (GLM) procedure, with all statistical models including diet group and baseline (pre-intervention) age, BMI, education, and the relevant baseline stress parameter (perceived stress scale or allostatic load) as independent variables.

Since the difference between the pre-intervention diet quality and the intervention diet quality (DGA; TAD) differed in magnitude for each subject, we hypothesized that these differences in diet quality shifts may possibly mediate the main effects of the intervention on 8-week change in perceived stress and allostatic load. Therefore, we also explored, separately, whether the change in dietary quality from the usual pre-intervention diet to the intervention diet mediated effects of the diet intervention on stress. Change in the Healthy Eating Index (ΔHEI) for each subject was used as a proxy for change in diet quality between the estimated pre-intervention diet and the assigned intervention diet (DGA or TAD). The change in total HEI and in each of the 12 HEI components were separately examined. In order to be considered as a mediator, the ΔHEI variable had to be significantly related to both assigned intervention diet (which was true of all such variables due to the design of the study) and to the outcome variable (change in perceived stress or allostatic load), controlling for baseline age, BMI, education, and the relevant baseline stress parameter. If both conditions were met, we included the ΔHEI variable in the statistical model described above and examined how its inclusion altered the regression coefficient for diet assignment, which represents the adjusted difference between the mean outcome variables of the two diets.

We also tested whether the associations (regression coefficients, ß) between ΔHEI and stress load differed between the two diet intervention groups by including a diet intervention group by ΔHEI interaction term in the model. In all cases tested, no significant diet group by ΔHEI interactions was observed and, therefore, the interaction term was removed from the final model. The final mediation model, which tested for main effects of the diet intervention (DGA vs. TAD) included the following independent variables: baseline age, BMI, education, the relevant baseline stress parameter (perceived stress scale or allostatic load), and the selected ΔHEI variable. In all cases, a *p*-value less than or equal to 0.05 was considered statistically significant.

## 3. Results

In this randomized control diet intervention, 8-week change in perceived stress did not significantly (*p* = 0.45) differ between the DGA (+0.53 ± 0.99) and TAD (−0.57 ± 0.99) groups (Table 1). Likewise, 8-week change in physiological stress load, as measured by the allostatic load index, did not significantly (*p* = 0.79) differ between the two diet intervention groups (DGA: +0.001 ± 0.26 vs. TAD: +0.105 ± 0.28).

**Figure 1:**
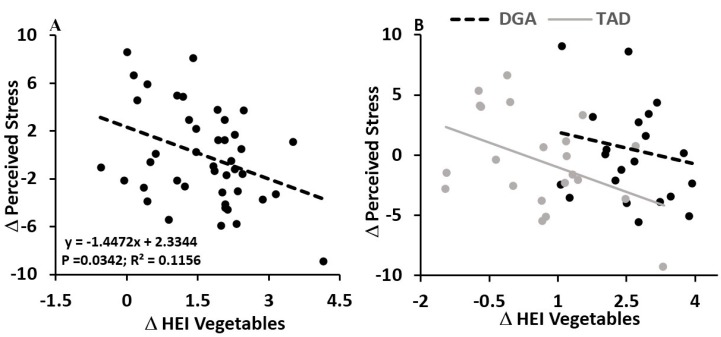
Independent of diet group, increasing the healthy eating index (HEI) for vegetable consumption associated with reductions in the 8 week perceived stress score (**A**). In the right hand figure (**B**), data and regression lines are presented for each diet group (DGA vs. TAD), showing that, for a given magnitude of delta HEI vegetables, the DGA group had a higher magnitude of perceived stress change. No statistical interaction between diet group and change in HEI vegetables was observed (*p* = 0.5062), suggesting the regression slopes between DGA and TAD groups were not different, with both demonstrating an inverse association between delta HEI vegetables and delta perceived stress. Data and regression lines presented in A and B were statistically adjusted for age, education, BMI, and pre-intervention perceived stress score.

However, we did find that the magnitude of change in diet quality (Delta Healthy Eating Index, HEI) between the estimated pre-intervention habitual diet and controlled intervention diet was related to both indices of stress load. While the total HEI in the DGA group significantly (*p* = 0.0001) improved (DGA: +31.59 ± 2.13 vs. TAD: −5.43 ± 2.13 points), on average, we found that an increase in HEI for vegetable consumption, specifically, was significantly (ß: −1.45 ± 0.66; *p* = 0.0342) associated with a reduction in perceived stress across the 8-week intervention, independent of diet group (Figure 1A). Moreover, this inverse association between change in perceived stress and change in HEI for vegetables appeared to mask, in part, effects of the diet intervention on perceived stress; when holding the change in HEI for vegetables constant by including this variable in the statistical model that evaluated effects of the diet intervention group, we found a significant (*p* = 0.0381) effect of the diet intervention. In the DGA group, perceived stress rose by 2.16 ± 1.20 points, compared to a decline (−2.21 ± 1.20 points) in this subjective index of stress perception in the TAD group (Figure 1B; Table 2). No other HEI component appeared to mediate the effects of the diet intervention on 8-week change in perceived stress.

**Figure 2:**
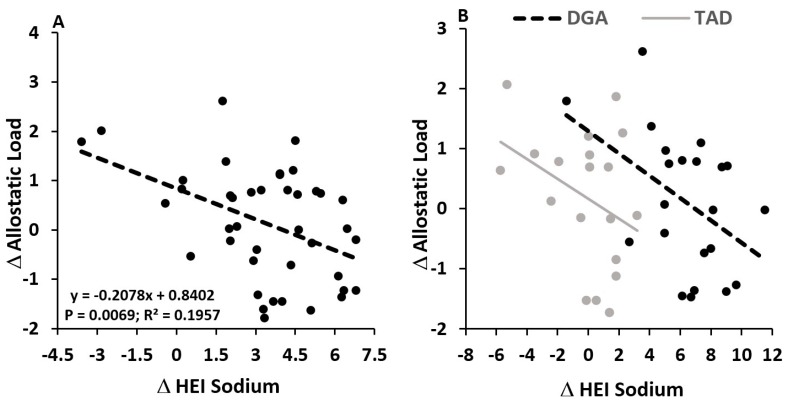
Independent of diet group, increasing the healthy eating index (HEI) for sodium consumption associated with reductions in the 8 week allostatic load (**A**). In the right hand figure (**B**), data and regression lines are presented for each diet group (DGA vs. TAD), showing that, for a given magnitude of delta HEI sodium, the DGA group had a higher magnitude of allostatic load change. No statistical interaction between diet group and change in HEI sodium was observed (*p* = 0.7909), suggesting that regression slopes between DGA and TAD groups were not different, with both demonstrating an inverse association between delta HEI sodium and delta allostatic load. Data and regression lines presented in A and B were statistically adjusted for age, education, BMI, and pre-intervention allostatic load.

**Figure 3:**
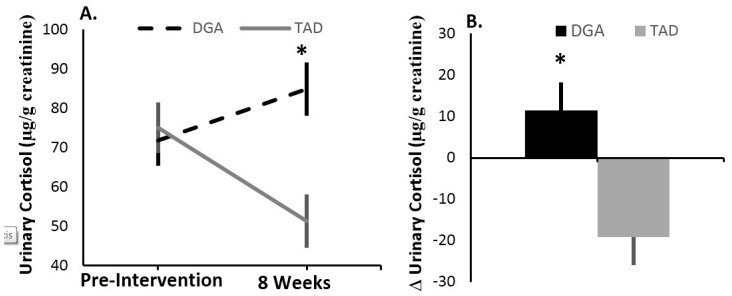
After controlling for the pre-intervention to intervention dietary shift in HEI for sodium, urinary cortisol concentration increased in the DGA group, while this stress marker declined in the TAD group. (**A**) shows pre-intervention and 8 week values (lsmean ± s.e.); (**B**) displays the delta (lsmean ± s.e.) between 8 week and pre-intervention values. All statistical models included diet group (DGA vs. TAD) and the covariates age, education, BMI, baseline stress marker, and the pre-intervention to intervention dietary change in HEI for sodium. * Indicates a significant (*p* < 0.02) difference between the DGA and TAD groups. DGA, Dietary Guidelines for Americans intervention group. TAD, Typical American Diet intervention group.

As with the self-reported index of stress perception, effects of the diet intervention on allostatic load appeared to be masked by the magnitude of departure in subjects’ intervention diet from their usual, pre-intervention diet. An increase in HEI for sodium consumption was significantly (ß: −0.21 ± 0.07; *p* = 0.0069) associated with a reduction in allostatic load across the 8-week intervention, independent of diet group (Figure 2A). Furthermore, when statistically holding the change in HEI for sodium constant, we found a significant (*p* = 0.0345) effect of the diet intervention, with the DGA diet associated with an increase (+0.70 ± 0.34) in allostatic load and the TAD diet associated with a decrease (−0.70 ± 0.38) in this biomarker of chronic stress load (Figure 2B; Table 2). No other HEI component impacted the effects of the diet intervention on 8-week change in allostatic load. However, similar to 8-week change in allostatic load, we found that, after controlling for change in sodium HEI, 8-week change in urinary cortisol concentrations was higher (*p* = 0.0147) in the DGA group (+11.47 ± 6.77 μg/g creatinine) compared to the TAD group (−19.12 ± 6.77 μg/g creatinine) (Figure 3). Moreover, like 8-week allostatic load changes, improvements in HEI for sodium were linearly associated with lower urinary cortisol changes over the 8-week intervention (ß: −3.34 ± 1.43; *p* = 0.0246). No other component of allostatic load was observed to associate with change in sodium HEI or differ between the DGA and TAD groups.

## 4. Discussion

### 4.1. Stress Load—Effects of the Diet Intervention

Stress is a key risk factor for disorders of mood and cognitive decline, and these effects of stress may, in part, result from the propensity of stress to promote poor nutrition habits and reduce adherence to health promoting diets, such as those based on the DGA. These dietary effects of stress can, therefore, significantly limit the utility of dietary recommendations aimed at treating or preventing diseases such as type 2 diabetes. On the other hand, it is also generally accepted that a healthy diet can help to manage stress and prevent stress-related disease such as depression. However, evidence based, specific dietary recommendations that address this issue do not exist. This study is the first of its kind to compare, in a randomized control trial, the effects of consuming DGA- or typical-American-based diets on perceived stress and stress load. Very little is known about how whole food diets, such as those based on the DGA, influence perceived stress and overall chronic stress load.

Our current findings suggest strong inverse associations between stress and improvements in diet quality (lower sodium and higher vegetable consumption), and the dampening effects of including less sodium and more vegetables in the diet appear to have been blunted or masked by possible stress-inductive effects of shifting to a diet of higher quality (DGA) for 8 weeks. Compared to the TAD, the DGA diet used in this study was, on average, associated with a higher total HEI [39], which is expected given that the study diet was designed to maximize adherence to the Dietary Guidelines for Americans [42]. Therefore, adopting and adhering to a diet of higher quality (DGA) for 8 weeks may have been generally more stressful in the absence of improvements in vegetable or sodium consumption. Furthermore and as intended in this study, body weight and composition were stable over the 8-week trial [39]. Therefore, it is extraordinary that these differences in stress between the DGA and TAD groups apparently resulted from differences related to diet quality, independent of major changes in body weight or composition.

There is some evidence that dietary restriction, independent of successful weight loss, can be stressful [58]. In part, this may be due to the chronic or repeated elimination of favorite foods [59,60]. Furthermore, careful monitoring of or attention to one’s diet has been thought by some to be a stressor [61] and has been shown to elevate perceived stress [58]. Dietary restraint, which is often associated with chronic dieting and repeated attempts to avoid preferred foods, even with unsuccessful weight loss, has been shown to be associated with elevations in stress markers and salivary and urinary cortisol, independent of BMI [62,63,64]. Alternatively, consumption of foods higher in added sugar and saturated fat have been shown to inhibit stress reactions [65,66]. Therefore, it is possible that differences in stress observed between DGA and TAD groups after controlling for changes in HEI for vegetables or sodium are a consequence of the TAD group consuming for 8 weeks a diet higher in saturated fat and added sugar. That is, it is possible decreases in HEI for vegetables or sodium, which would be presumed to elevate stress, may have been masked by the stress-reducing effects of consuming a diet higher in foods that have been shown to dampen stress reactions. Both possibilities could have contributed to the observed differences in stress between the diet intervention groups.

### 4.2. Diet Quality Effects—Vegetables and Perceived Stress

Generally, there are reports showing inverse associations between diet quality (e.g., total HEI) and mood and emotional state [67,68,69,70]. There is also a recent report by Lutz et al. [40] demonstrating that a higher HEI increased the odds of being characterized as stress resilient. More specifically, previously published reports, primarily from cross-sectional studies, corroborate our findings of an inverse relationship between improvements in vegetable consumption and stress, mood, and psychological wellbeing. The DGA diet used in this study contained more than double the total vegetable content of the TAD diet. Of this, there was much higher dark green vegetable (233% more), red and orange vegetables (267% more), and starchy vegetable (200% more) content. In addition, there was also more than double the amount of fruits, which included four times more whole fruit in the DGA vs. TAD diet. Fruit and vegetable consumption has been shown to be associated with lower self-reported perceived stress [71,72,73,74,75], reduced risk of major depression [76], better psychological wellbeing [77], previous diagnosis of a mood disorder and anxiety disorder, and significantly lower perceptions of poor mental health [78]. Our results are unique in that they show an inverse association between the magnitude of improvement in vegetable consumption and prospective increases in perceived stress. Moreover, and based on this relationship, one of the possible key benefits of adhering to the Dietary Guidelines for vegetables in the context of dieting may be its stress-buffering effect. Given that dietary and weight relapse is frequent [61], with psychological stress being a major factor that increases vulnerability to relapse, this apparent stress damping effect of including more vegetables in the diet may help to prevent dietary relapse and improve DGA adherence. More studies are needed to test the psychological effects of including vegetables in whole food diets aimed at reducing chronic disease.

We can only speculate as to why improvements in vegetable consumption associated with lower perceived stress at the end of the study. One possibility might be that micronutrients in vegetables, such as the B vitamins and their metabolites [79,80,81], vitamin C [82], zinc [83], magnesium [84], and calcium [85], facilitated healthy regulation of brain pathways critical to maintaining a healthy mood and emotional state. These micronutrients are known to support healthy regulation of mood, optimal cognitive function, neurotransmitter synthesis and activities involved in healthy regulation of stress responsiveness, and antioxidant functions in the brain. Bottiglieri et al. reported that approximately one-third of the most severe presentations of depression were associated with folate deficiency, as observed by red cell folate concentration. They suggest that this is a product of impaired methylation of homocysteine to methionine by S-Adenosyl methionine (SAM) methylation, thus translating into impaired activity of the neurotransmitters serotonin, dopamine, and noradrenaline, which play key roles in stress, mood, and emotion regulation [86,87].

Alternatively, because subjects in this study were provided with all of their meals, it is possible that individuals who may have ordinarily had limited access to vegetables due to financial- or time-related constraints may have felt less stress about purchasing and preparing fresh vegetables. Vegetable consumption in particular is positively correlated with socioeconomic status [88,89,90], and this meal-fed intervention may have reduced this known barrier to vegetable consumption. Enabling easier access to and consumption of more vegetables in this study may have also provided a greater sense of self-control or a sense of accomplishment, possibly having positive effects on overall mood and emotional state. Future studies are warranted to better understand the mechanisms, particularly in the context of whole food diets, by which vegetable consumption affects psychological health.

### 4.3. Diet Quality Effects—Sodium and Allostatic Load

There are relatively few reports on the relationship between diet quality and allostatic load, but it was previously suggested that poor diet likely contributes to increased allostatic load [91]. A diet characterized by foods high in sodium was shown to associate with elevated allostatic load [92], and Mattei et al. showed that adherence to an American-Heart-Association-based diet associated with lower allostatic load and urinary cortisol [93]. There also exists some evidence that increases in sodium or salt load can raise urinary cortisol [94,95,96], while restricting sodium appeared to reduce urinary cortisol [97]. Overall, these reports corroborate our findings showing an association between improvements in HEI for sodium and lower physiological stress load.

In this study, improvements in dietary sodium, as indicated by positive changes in HEI for sodium, were associated with lower 8-week physiological stress load. Moreover, lower urinary cortisol concentrations were similarly associated with greater improvements in HEI for sodium. Our results suggest that this relationship may have also mediated effects of the diet intervention on this physiological marker of chronic stress load. Therefore, a more marked intervention-related improvement from subjects’ regular (pre-intervention) habits in sodium consumption appeared to inhibit physiological symptoms of chronic stress. When we statistically controlled for these interindividual differences in departure from the pre-intervention diet, it became evident that, compared to the TAD, being on the DGA diet may have elevated overall physiological stress load; reductions in sodium consumption (improved HEI for sodium) appeared to mask this apparent stress-inductive effect, resulting in no differences in allostatic load between the DGA and TAD groups.

The amount of cortisol in the urine is considered as a noninvasive, integrated measure of Hypothalamic-Pituitary-Adrenal (HPA) activity and cortisol exposure [98,99]. However, altered urinary cortisol concentrations can reflect multiple factors, including changes in cortisol metabolism, changes in hypothalamic–pituitary–adrenal activity, and altered filtration/excretion. We attempted to adjust for differences in excretion rates by using the cortisol:creatinine ratio, thereby providing a standardized estimate of cortisol to urine output. Regardless, increased urinary cortisol:creatinine suggests greater exposure to cortisol. As with the associations between vegetable consumption and perceived stress, we can only speculate on possible mechanisms linking improvements in sodium consumption (increases in HEI for sodium) and lower urinary cortisol. Reductions in sodium consumption and circulating concentrations affect the renin–angiotensin system, and a high salt diet inhibits this system. There is also an inverse relationship between angiotensin II and pituitary secretion of adrenocorticotropic hormone (ACTH) [100], which stimulates adrenal synthesis and secretion of cortisol. Therefore, it is possible that improvements in sodium consumption led to lower urinary cortisol as a result of elevated angiotensin activity at the pituitary with suppression of ACTH secretion. It is also possible that the observed association between sodium consumption and urinary cortisol in this study resulted from alterations in activity of key enzymes involved in cortisol metabolism, such as 5β-reductase and 11β-HSD; restrictions in sodium consumption have been shown to alter these enzymes in directions that enhance catabolism of cortisol [97]. These explanations are only speculative, but our results warrant mechanistic studies to determine how changes in dietary sodium link to alterations in cortisol.

Given the known detrimental health risks of chronic stress load, in part through heightened exposure to cortisol, these findings may have important clinical implications. Increases in chronic stress and cortisol have been linked to elevated risk for central obesity, metabolic syndrome, cardiovascular disease, type 2 diabetes, depression, and other disorders of the brain [101,102]. Therefore, adherence to dietary sodium guidelines may help to prevent unhealthy stress reactions and its effects on mental and metabolic health. Furthermore, our findings suggest that, in the context of dieting, adherence to sodium consumption guidelines may be critical for minimizing the stress of restricting favorite, highly palatable comfort foods [65,66,103]. Chronic stress and overexposure to the glucocorticoid hormone, cortisol, can increase preference for and drive to eat highly palatable, nutrient-scarce foods [104], thereby reducing in some persons their capacity for sustaining significant and durable improvements in diet quality. Our findings suggest that limiting sodium intake as part of a whole food diet may help minimize overexposure to cortisol and its effects of increasing hunger for comfort foods.

### 4.4. Overall Implications

This study provides further evidence for the health benefits of maximizing vegetable and minimizing sodium consumption. We further extend a growing body of support for the beneficial effects of a healthy diet on mental health. Our findings provide new information that might help to inform on key food groups that facilitate improved adherence to healthy changes in diet, which are often fleeting. There were other differences between the two diets that did not directly translate into direct implications for the stress markers measured by this study. For example, there was more than twice the amount (155% more) whole grain content and approximately half as much (58%) refined grains in the DGA diet compared to the TAD diet. In addition, the DGA diet contained 20% less meat, fish, poultry, eggs, nuts but 83% more than double (120% more) of the dairy content than the TAD diet. Finally, the DGA diet contained 46% more oil and 55% less solid fat and added sugar than the TAD diet. However, these differences did not translate into direct differences in stress between the two subject groups. Further research should probe further into whether the same relationship between high vegetable and low sodium consumption and measures of stress can be observed when such differences do not exist. As such, our results support new and key target areas for improving nutrition and disease prevention messaging.

### 4.5. Limitations

To our knowledge, this is the first controlled-feeding trial comparing psychological and physiological stress effects of a DGA-based diet and a more representative American diet. However, we acknowledge that there are limitations associated with this trial, as previously reported [39]. The relatively short duration of 8 weeks in this study may not represent effects or lack thereof over a longer timeframe. Another potential limitation is the relatively small sample size used in this intervention. It is also important to note that this study was conducted in women and results cannot necessarily be extrapolated to apply to men or to people with normal glucose or lipid values.

## Figures and Tables

**Table 1:** * Effects of diet intervention on perceived stress and allostatic load.

Stress Marker	Diet	Pre-Intervention	8 Weeks	8-Week Change
Perceived Stress	DGA	10.92 ± 1.47	13.23 ± 1.00	0.53 ± 1.00
	TAD	14.48 ± 1.40	12.13 ± 1.00	−0.57 ± 1.00
Allostatic Load	DGA	2.42 ± 0.37	2.42 ± 0.26	0.001 ± 0.26
	TAD	2.41 ± 0.40	2.52 ± 0.28	0.105 ± 0.28

* All statistical models included diet group (DGA vs. TAD) and the covariates age, education, Body Mass Index (BMI), and baseline stress marker. Pre-intervention data represent least-squared means ± standard errors after adjusting for age, education, and BMI. Eight-week data represent least-squared means ± standard errors after adjusting for age, education, BMI, and pre-intervention stress marker. No statistical differences between DGA and TAD were observed. DGA, Dietary Guidelines for Americans Diet. TAD, Typical American Diet.

**Table 2:** * Mediation effects of changing diet quality (HEI) on perceived stress and allostatic load.

Stress Marker	Diet	Pre-Intervention	8 Weeks	8-Week Change
** Perceived Stress	DGA	10.92 ± 1.47	14.87 ± 1.20 **^a^**	2.16 ± 1.20 **^b^**
	TAD	14.48 ± 1.40	10.50 ± 1.20	−2.21 ± 1.20
*** Allostatic Load	DGA	2.42 ± 0.37	3.11 ± 0.34 **^a^**	0.70 ± 0.34 **^b^**
	TAD	2.41 ± 0.40	1.71 ± 0.38	−0.70 ± 0.38

* All statistical models included diet group (DGA vs. TAD) and the covariates age, education, BMI, and baseline stress marker. ** Statistical model also included change in the Healthy Eating Index (HEI) for vegetable consumption. *** Statistical model also included change in HEI for sodium consumption. Pre-intervention data represent least-squared means ± standard errors after adjusting for age, education, and BMI. Eight-week data represent least-squared means ± standard errors after adjusting for age, education, BMI, pre-intervention stress marker, and, where indicated (see asterisks, above), change in HEI. **^a^** For each stress marker and compared to the TAD group, an “a” superscript indicates a significantly (*p* < 0.05) higher 8-week level of stress in the DGA group. **^b^** For each stress marker and compared to the TAD group, an “b” superscript indicates a significantly (*p* < 0.05) higher 8-week change in level of stress in the DGA group. DGA, Dietary Guidelines for Americans Diet; TAD, Typical American Diet.
